# Addiction to Smartphone Use in Smokers Diagnosed with Type 2 Diabetes in Jordan: Are Their Medications Involved?

**DOI:** 10.3390/healthcare12242559

**Published:** 2024-12-19

**Authors:** Omar Gammoh, Mervat Alsous, Mariam Al-Ameri, Sereene Al-Jabari, Lana Sbitan, Jafar Alsheyyab, Sa’ed Zeitoon, Suzan Hanandeh, Alaa A. A. Aljabali, Hayam Ali AlRasheed, Sireen Abdul Rahim Shilbayeh

**Affiliations:** 1 Department of Clinical Pharmacy and Pharmacy Practice, Faculty of Pharmacy, Yarmouk University, Irbid 21163, Jordan; mervat.alsous@yu.edu.jo (M.A.); m.alameri@yu.edu.jo (M.A.-A.); 2 Faculty of Pharmacy, The University of Jordan, Amman 11942, Jordan; serenenedal2001@gmail.com; 3 Faculty of Medicine, The Hashemite University, Zarqa 13133, Jordan; lanay.sbitan@gmail.com (L.S.); jafar@hu.edu.jo (J.A.); saedm@hu.edu.jo (S.Z.); 4 Prince Hamzeh Hospital, Amman 11118, Jordan; suzan_hanandeh@yahoo.com; 5 Department of Pharmaceutics and Pharmaceutical Technology, Faculty of Pharmacy, Yarmouk University, Irbid 21163, Jordan; alaaj@yu.edu.jo; 6 Department of Pharmacy Practice, College of Pharmacy, Princess Nourah bint Abdulrahman University, Riyadh 11671, Saudi Arabia; haalrasheed@pnu.edu.sa (H.A.A.); ssabdulrahim@pnu.edu.sa (S.A.R.S.)

**Keywords:** diabetes, smartphones, risk factors

## Abstract

Background/Objectives: The prevalence of type 2 diabetes and smoking is increasing in developing countries and is associated with deteriorated health outcomes. Also, addiction to smartphone use is an alarming behavior that can be associated with clinical factors. This study aimed to determine the prevalence and clinical correlates of smartphone addiction in smokers with T2DM in Jordan, with a particular focus on the role of medications. Methods: This cross-sectional study recruited patients from Prince Hamza Hospital, Jordan, according to pre-defined criteria. Besides demographics and clinical information, this study used the validated Arabic version of the Smartphone Addiction Scale to assess addiction to smartphones and a multivariable regression analysis to identify the correlates of smartphone addiction. Results: Data analyzed from 346 patients revealed that 117 (33.8%) of these participants reported addiction to smartphones. Patients who had been diagnosed with T2DM for less than five years (aOR = 3.30; 95% CI = 1.43–7.60), who were “employed” (aOR = 8.85; 95% CI = 2.20–35.64), and who were “retired” (aOR = 11.46; 95% CI = 2.72–48.23) all reported a significantly (*p* < 0.05) higher odds of smartphone addiction. In contrast, patients on “sulfonylurea” (aOR = 0.18; 95% CI = 0.06–0.53); “metformin” (aOR = 0.19; 95% CI = 0.06–0.66), and “gabapentin” (aOR = 0.16; 95% CI = 0.04–0.67) and those with “comorbid hypertension” (aOR = 0.15; 95% CI = 0.06–0.38) had a significantly (*p* < 0.05) lower odds of smartphone addiction. Conclusion: These alarming results require adequate action from the health authorities to raise awareness of adopting positive behaviors that could improve the well-being of this high-risk population.

## 1. Introduction

Diabetes is one of the most common chronic illnesses globally, especially type 2 diabetes mellitus (T2DM), which affected approximately 537 million adults in 2021 and is expected to affect 643 million by 2030, according to the International Diabetes Federation (IDF) [1]. The long-term complications of T2DM include kidney failure, neuropathy, and cardiovascular disease, which put a significant strain on patients and healthcare systems [2]. Over the past 20 years, Jordan has seen a notable increase in T2DM cases, similar to many other Middle Eastern nations [3]. According to a recent systematic review and meta-analysis that studied Middle Eastern type 2 diabetes prevalence, the maximum reduction in the risk of acquiring diabetes should be a top priority for health, as evidenced by the concerning patterns and expanding trends in diabetes for the last 20 years in the examined area [4]. It is well established that this increasing trend of T2DM is a consequence of lifestyle, environmental, and hereditary variables, in particular poor eating habits, smoking, and sedentary lifestyles [5].

In addition, excessive use of smartphones was associated with low physical exercise, overeating, and therefore obesity [6,7]. This is a concern in people with chronic medical conditions, such as type 2 diabetes, and this could exacerbate the clinical picture [8]. The inability to control one’s use of smartphones is known as smartphone addiction, and it has been linked to several mental and physical health problems [9]. The overuse of smartphones by T2DM patients can worsen their blood sugar control by reinforcing sedentary behaviors, interfering with their sleep cycles, and raising their stress levels [10]. Moreover, it has been connected to a decline in physical activity, which is an important lifestyle intervention in the treatment of type 2 diabetes [11].

According to several studies, diabetes and smoking interacted to increase the risk of cardiovascular (CVD) events, and smoking in the past or present reduced the protective effects of risk factor management on CVD risk in patients with diabetes [12,13,14]. Diabetes in Jordan has been made worse by the rising smoking rate. According to recent figures, although 70% of adults in Jordan have access to nicotine replacement therapy, however, smoking is still a widely spread habit [15].

In addition, smartphone use is a nexus that warrants investigation; this behavior can be associated with several overlooked psychiatric, demographical, and clinical variables.

Therefore, this study aimed to determine the prevalence and clinical correlates of smartphone addiction in smokers with T2DM in Jordan, with a particular focus on the role of medications.

## 2. Materials and Methods

### 2.1. Study Design and Settings

**Figure 1:**
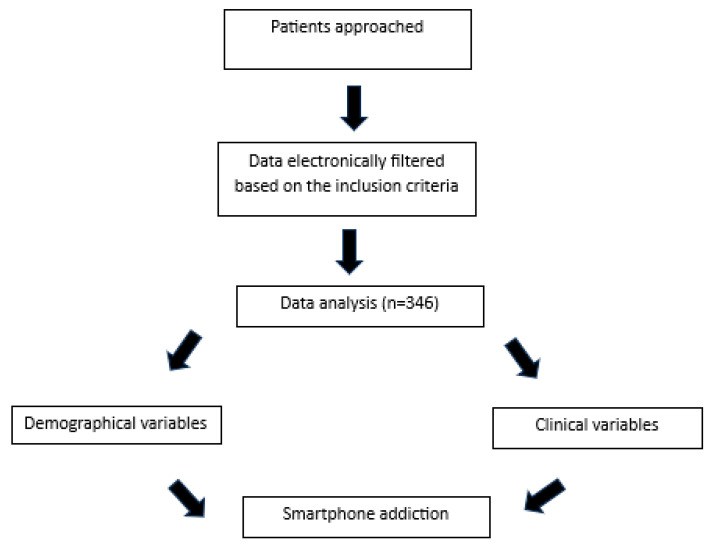
Study flow chart.

This cross-sectional study recruited patients attending the outpatient clinics in Prince Hamza Hospital. The researchers encountered the potential participants in the waiting area before they visited a physician and explicitly explained to them the aim and the protocol of the study. Immediately, a link was sent to all willing participants that directed them to the study questionnaire using Google Forms. One of the researchers assisted the patients with any technical issues. The study instrument filtered outpatients who did not fulfill the inclusion criteria by terminating the survey. In addition, incomplete data could not be submitted. The data collection stopped upon receiving a representative sample size, as in our previous studies [16]. This study was approved by the Yarmouk University IRB Committee (692/2023). Please refer to the flow chart in Figure 1 below.

### 2.2. Inclusion Criteria

Patients diagnosed with T2DM who identified themselves as smokers, attended the outpatient clinics at Prince Hamza Hospital, were aged above 18 years old, and filled out the data in collaboration with the research team members were included in this study.

### 2.3. Study Instrument

The researchers used a well-structured questionnaire to achieve the objectives of this study. The participants’ demographic and clinical information was captured in detail. For instance, the participants answered questions about their age, sex, marital status, and current employment status. In addition, the participants reported whether they were diagnosed with other long-term diseases, namely hypertension, dyslipidemia, or both. Also, the participants reported the duration of their diagnosis with T2DM, their disease control status, the diabetes therapy(s) they received, their neuropathy severity based on the Arabic validated (Cronbach’s α = 0.79) version of the LANNS scale [17]—which generates a maximum score of 24, with a score of 12 or more identifying significant neuropathic pain—and the medications they currently used to manage neuropathy.

### 2.4. The Outcome Variable: Addiction to Smartphones

This variable was screened using the validated and reliable Arabic version of the Smartphone Addiction Scale. This self-administered scale comprises ten questions (focused on emotional dependency and the time spent on smartphones) rated against Likert-scale answers with a score range between 1 and 6. The scale yields a score ranging from “0” to “60”; according to the literature, a score ≥39 is used to identify behavior of addiction to smartphones, as demonstrated in a Jordanian cohort [18,19]. A confirmatory factor analysis (CFA), a reliability analysis, and concurrent and convergent validity assessments were performed. The scale exhibited adequate internal consistency and test–retest reliability (Cronbach’s α = 0.79; test–retest reliability = 0.83) and good concurrent and convergent validity [20].

### 2.5. Data Analysis

The patients’ information was displayed as frequencies and percentages. The association between the patients’ factors and their addiction to smartphones was examined using a chi-square analysis because these variables were categorical. Next, variables showing *p* < 0.10 were used to build a step-wise backward multivariable binary regression model, with only variables at *p* < 0.05 kept in the final model. The confidence intervals were set at 95% and significance at *p* < 0.05.

## 3. Results

### 3.1. Study Sample Characteristics

Analysis of the data of the 346 T2DM patients that met the inclusion criteria revealed that 179 (51.7%) were male, 175 (50.6%) were above 60 years old, 180 (52%) were married, and 112 (32.4%) were retired. Clinically, 233 (67.3%) were diagnosed with hypertension, 231 (66.8%) reported elevated cholesterol, and 215 (62.1%) reported hypertension and elevated cholesterol. Furthermore, 242 (69.9%) reported having a diagnosis of T2DM for more than 5 years, and 233 (67.3%) met the threshold score for significant neuropathy according to the LANNS scale. In addition, 279 (80.6%) patients were on metformin, 157 (45.4%) were on metformin, 124 (35.8%) were on insulin, 93 (26.9%) were on carbamazepine, 91 (26.3%) were on gabapentin, and 284 (82.1%) were on over-the-counter analgesics, such as acetaminophen and non-steroidal anti-inflammatory drugs. Please refer to Table 1 for further details.

### 3.2. Prevalence and Correlates of Smartphone Addiction

According to the Arabic version of the smartphone addiction scale, 117 (33.8%) participants reported being above the threshold for addiction. For example, 45% responded “I agree” and 17% responded “I strongly agree” to the question “Missing planned work due to smartphone use”; similarly, 41% responded “I agree” and 16% responded “I strongly agree” to the question “Having a hard time concentrating in class, while doing assignments, or while working due to smartphone use”. In addition, 37% responded “I agree and (14%) responded “I strongly agree” to the question “The people around me tell me that I use my smartphone too much”. According to the chi-square analysis, all of the independent factors showed a significant association with smartphone addiction at the univariate level, except gender. All of these factors were used to inform the multivariable backward step-wise model.

The final model for the multivariable logistic regression analysis included the following correlates with smartphone addiction: “T2DM disease duration”, “employment status”, “comorbid hypertension”, “sulfonylurea”, “metformin”, and “gabapentin.” Please refer to Table 2 for further details. Patients who had been diagnosed with T2DM for less than five years (aOR = 3.30; 95% CI = 1.43–7.60), who were “employed” (aOR = 8.85; 95% CI = 2.20–35.64), and who were “retired” (aOR = 11.46; 95% CI = 2.72–48.23) all reported a significantly (*p* < 0.05) higher odds of smartphone addiction. In contrast, patients who were on “sulfonylurea” (aOR = 0.18; 95% CI = 0.06–0.53), “metformin” (aOR = 0.19; 95% CI = 0.06–0.66), and “gabapentin” (aOR = 0.16; 95% CI = 0.04–0.67) and who were diagnosed with “comorbid hypertension” (aOR = 0.15; 95% CI = 0.06–0.38) had a significantly (*p* < 0.05) lower odds of smartphone addiction. Please refer to Table 2 for further details.

## 4. Discussion

This study provides a better understanding of the prevalence and correlates of smartphone addiction in a particular sample of patients with T2DM of Jordan. Indeed, its findings revealed a smartphone addiction prevalence of 33.8%, which is higher than that reported in the general population in various studies [21]. This reflects the vulnerability of T2DM patients to addictive smartphone use, likely due to their chronic illness, psychological burden, and increased dependency on digital tools in managing their disease.

The clinical and demographic correlation analyses performed for the characteristics of the study sample revealed several important demographic and clinical features in this population. Interestingly, the rate of smartphone addiction was not confined to younger individuals; equally, 51.7% of the patients were male, while 50.6% were over 60 years of age.

Therefore, these findings challenge the general view that young people are the most susceptible to smartphone addiction and indicate that even older adults with T2DM can be highly vulnerable. This may be attributed to the increasing use of smartphones among older populations for health-related purposes, including managing one’s condition through digital tools and maintaining social connections later in life.

One published report has noted that more than half of its participants were married (52%) or retired (32.4%) [22]. Retired people may use a smartphone to compensate for poor social connections and loneliness. Most retirements lead to social isolation, and the ease of maintaining relationships through smartphones may contribute decisively to the rates of addiction observed.

This might be because smartphones are no longer just a tool for work but also a source of recreation or stress relief for employed people. This may explain why the odds of addiction were higher in this group. This underlines the present findings that both work-related stress and social isolation can drive the addictive use of smartphones [23,24].

### 4.1. Medications and Current Health Conditions

The findings of this study involve the relationship between smartphone addiction and the use of medication. Sulfonylurea (aOR = 0.18), metformin (aOR = 0.19), and gabapentin (aOR = 0.16) medications also showed a lower odds of developing smartphone addiction, at least in our study sample. This could indicate a protective effect of these medications, although its mechanisms are not immediately clear.

One would imagine that the patients on these medications may have their blood sugar and symptoms under greater control, with them thereby having less of a need for distraction or coping mechanisms, such as smartphone use. Metformin plays a central role in the stabilization of blood glucose, which should provide at least some positive effects on mood and cognition and thus reduce the psychological motivations for excessive smartphone use [25,26,27].

This is interesting considering that gabapentin is mainly used to treat neuropathic pain. It is possible that in patients in whom neuropathy is somewhat improved, there is less need for distractions, such as the use of smartphones, because of their general level of discomfort. This could also be further investigated longitudinally through studies assessing how the management of symptoms through medication influences behavioral outcomes such as smartphone addiction. 

### 4.2. T2DM Duration and Addiction to Smartphones

The duration of T2DM was one of the strongest correlates of smartphone addiction in the current study. Patients diagnosed with T2DM less than five years ago had a significantly higher likelihood of smartphone addiction, with an aOR of 3.30. This finding is probably due to the information-seeking behavior of recently diagnosed patients.

We assume that newly diagnosed patients may overuse their smartphones to access health information to alleviate their sense of anxiety. However, this may eventually develop into addictive behavior patterns in patients, as they seek to use these devices to lower their stress resulting from disease management.

This is in line with the available literature, which correlates increased adoption of smartphones with the early stages of a chronic condition’s management [28]. However, in cases of a longer disease duration, patients may have already established routines for managing their disease after five years and have become less dependent on smartphones. In addition, this group may have also developed confidence in managing their disease and have experienced reduced psychological distress and information-seeking, which turns newly diagnosed patients into people addicted to their smartphones.

### 4.3. Comorbidity and the Utilization of Smartphones

The study population was highly comorbid with hypertension (67.3%) and elevated cholesterol (66.8%). However, the patients with comorbid hypertension were significantly less likely to develop smartphone addiction, with an adjusted odds ratio of 0.15. This contradicts the expectation that multiple chronic conditions could lead to increased addiction because of an increased reliance on smartphones to manage their health.

One possible reason for this negative correlation may be that their worse conditions, such as hypertension, made these patients more cautious about their physical health in their better self-care practices, hence them using their smartphones less. This group may also have been more concerned about lifestyle changes, such as regular exercise and dietary changes, which could have indirectly helped to reduce their screen time. Conversely, medical care and monitoring for comorbidities are more frequent in patients with hypertension; therefore, they may have less need to seek out health-related information through their smartphones.

This protective effect, although it was seen in our study sample, not being generalizable, calls for further investigation, especially regarding whether specific lifestyle interventions or behavioral counseling in the management of hypertension contributes to lower smartphone addiction.

### 4.4. Employment Status and Smartphone Addiction

Employment status was a significant predictor of smartphone addiction, with both employed (aOR = 8.85) and retired (aOR = 11.46) participants having a higher odds than the unemployed. This higher number of employed people could be because working people increasingly use their smartphones on account of work, with an increasing trend towards work-from-home and hybrid models.

These patients’ employment status may have also led to higher levels of work-related stress, which again may have acted as a motivational factor for escapist behaviors such as smartphone addiction. This possibility is supported by published reports [29,30,31]. However, retirees may use their smartphones due to their unstructured activities, such as using social media platforms to reach out to family and friends.

This suggests that targeted interventions are necessary to encourage retired persons to use smartphones healthily, especially retirees with chronic conditions such as T2DM, who are more susceptible to their excessive use, either due to social isolation or boredom.

Cultural considerations in the use of smartphones: Given that this study was conducted within an Arab population, cultural elements may impinge on the prevailing patterns of smartphone use.

Additionally, the fast-growing application of mobile health technologies across the Arab region might further increase engagement in smartphone use among people with chronic conditions, such as T2DM. This increased engagement in digital health might lead to a new addiction [32] if it is not complemented by appropriate guidelines for its healthy use [33].

However, more studies are needed to ascertain how cultural factors influence smartphone use in the Arab population concerning the management of chronic diseases. Such dynamics will provide insight into the culturally appropriate development of interventions aimed at reducing addiction to smartphones while ensuring healthy usage of digital tools in the management of T2DM.

### 4.5. Clinical Implications and Recommendations

These findings have significant clinical relevance to informing targeted interventions, at least among Jordanian people diagnosed with T2DM. For example, efforts can be made in retired patients to increase their awareness of the potential educational benefits of the proper use of smartphones for diabetes.

Healthcare professionals should be aware of the high prevalence of smartphone addiction among T2DM patients, especially those who are newly diagnosed or employed. Smartphones can serve as useful tools for helping individuals to cope with their chronic conditions, but their excessive use may have negative implications for patients’ psychological well-being and glycemic control. Clinicians should consider routine screening for smartphone addiction in T2DM patients and provide appropriate guidance regarding the balanced use of smartphones. Also, in collaboration with psychiatrists, structured programs to reduce social isolation through in-person interactions could be implemented.

At the national level, efforts can be put together with stakeholders and public health policymakers to foster the healthy use of technology among patients. For example, healthcare practitioners can also be involved in the education of patients on the potential risks of smartphone addiction regarding the management of chronic diseases. Mindfulness-promoting interventions related to smartphone use could be designed to foster other modes of coping mechanisms, such as physical activity, and techniques to reduce stress, which would provide a protective effect against addiction in this population.

### 4.6. Study Strengths

The research topic, the sample type and its representative size, the validated scales, and the robust data analysis were all strengths of the current study.

### 4.7. Study Limitations and Future Research

This study has several limitations. The research design is cross-sectional; therefore, a temporal relationship between the established correlates and smartphone addiction cannot be determined. This study should be followed by longitudinal studies to establish the temporal relationship between these factors and the actual behavioral changes over time among patients with T2DM. However, the present study was conducted in a specific population using the Arabic version of the Smartphone Addiction Scale, which may restrict the generalization of its findings to other populations. Further studies should assess the consistent patterns of smartphone addiction among T2DM patients in other cultural, socioeconomic, physical-activity-related, dietary-pattern-related, and psychological contexts. Moreover, this study did not investigate the purposes for which smartphones were used, such as health applications, compared to social networking, which may have provided further explanations of the nature of addiction among the population that this sample represented. In addition, the scale used to assess smartphone addiction does not currently correspond to diagnostic criteria—for example, the ICD-10, ICD-11, or DSM-5. Future research is warranted to study the varied uses of smartphones and their contributions to addiction risk and whether targeted interventions can help to promote healthy smartphone behaviors among T2DM patients. In conclusion, this study established a high prevalence of smartphone addiction among patients with T2DM and several important correlates, such as disease duration, employment status, medication use, and comorbidities. These findings support the complex interplay between chronic disease management, medication use, and digital technology engagement. Concomitantly, the importance of smartphones in diabetes care requires health providers to balance the benefits of these tools with the risks of their addictive use. Future research is needed to develop targeted interventions that encourage healthy smartphone use while supporting effective chronic disease management.

## 5. Conclusions

These findings support the complex interplay among chronic disease management, medication use, and digital technology engagement among Jordanian people with T2DM. This should go beyond the mere clinical management of diabetes. Concomitantly, the importance of smartphones in diabetes care requires health providers to balance the benefits of these tools with risks of their addictive use. Future research is needed to develop targeted interventions such as support groups, clubs, and individual sessions that can encourage healthy smartphone use while supporting effective chronic disease management.

## Figures and Tables

**Table 1:** The association between the patients’ demographic and clinical factors and addiction to smartphones (*n* = 346).

Factor	Category	Below the Addiction Threshold (*n* = 229, 66.2%)	Met the Addiction Threshold (*n* = 117, 33.8%)	Chi-Square Score	*p*
		*n* (%)	*n* (%)		
Gender	Male	121 (52.8)	58 (49.6)	0.33	0.57
	Female	108 (47.2)	59 (50.4)		
Age	<60 years	54 (23.6)	117 (100.0)	180.91	<0.001
	≥61 years	175 (76.4)	0 (0.0)		
Marital status	Single	4 (1.7)	55 (47.0)	137.49	<0.001
	Married	124 (54.1)	56 (47.9)		
	Divorced	95 (41.5)	2 (1.7)		
	Widow	6 (2.6)	4 (3.4)		
Employment	Does not work	35 (15.3)	65 (55.6)	92.42	<0.001
	Works	85 (37.1)	49 (41.9)		
	Retired	109 (47.6)	3 (2.6)		
Comorbid hypertension?	No	17 (7.4)	96 (82.1)	196.09	<0.001
	Yes	212 (926)	21 (17.9)		
Comorbid high cholesterol?	No	24 (10.5)	91 (77.8)	158.04	<0.001
	Yes	205 (89.5)	26 (22.2)		
Comorbid hypertension and high cholesterol?	No	31 (13.5)	100 (85.5)	170.31	<0.001
	Yes	198 (86.5)	17(14.5)		
T2DM diagnosis for	<5years	37 (16.2)	67 (57.3)	62.24	<0.001
	≥5 years	192 (83.6)	50 (42.7)		
T2DM controlled?	Yes	68 (29.7)	87 (74.4)	70.21	<0.001
	No	137 (39.8)	14 (14.5)		
	Not sure	24 (10.5)	13 (11.1)		
Metformin?	No	7 (3.1)	60 (51.3)	115.33	<0.001
	Yes	222 (96.9)	57 (48.7)		
Sulfonylurea?	No	78 (34.1)	111 (94.9)	115.53	<0.001
	Yes	151 (65.9)	6 (5.1)		
Insulin?	No	189 (82.5)	33 (28.2)	99.39	<0.001
	Yes	40 (17.5)	84 (71.8)		
Significant neuropathy according to the LANNS?	No	23 (10.0)	90 (76.9)	157.48	<0.001
	Yes	206 (90.0)	27 (23.1)		
Carbamazepine?	No	146 (63.8)	107 (91.5)	30.22	<0.001
	Yes	83 (36.2)	10 (8.5)		
Gabapentin?	No	141 (61.6)	114 (97.4)	51.38	<0.001
	Yes	88 (34.4)	3 (2.6)		
OTC analgesics for neuropathy?	No	8 (3.5)	54 (46.2)	95.81	<0.001
	Yes	221 (96.5)	63 (53.8)		

T2DM: type 2 diabetes mellitus; LANSS: Leeds Assessment for Neuropathic Symptoms and Signs; OTC: over-the-counter.

**Table 2:** Association between the independent variables and the dependent variable (addiction to smartphones) using multivariable binary logistic regression.

Independent Variable	B	Wald	aOR	95% CI	*p*-Value
T2DM diagnosis < 5 years	1.19	7.89	3.30	1.43–7.60	0.005
Sulfonylurea	−1.71	9.60	0.18	0.06–0.53	0.002
Metformin	−1.61	6.95	0.19	0.06–0.66	0.008
Employed	2.18	9.42	8.85	2.20–35.64	0.002
Retired	2.43	11.07	11.46	2.72–48.23	0.001
Comorbid hypertension	−1.87	16.27	0.15	0.06–0.38	0.001
Gabapentin	−1.81	6.23	0.16	0.04–0.67	0.013

T2DM: type 2 diabetes mellitus, aOR: adjusted odds ratio, CI: confidence interval.

## Data Availability

The data will be made available by the corresponding author upon request.
